# Consistency of Leadership in Shoals of Mosquitofish (*Gambusia holbrooki*) in Novel and in Familiar Environments

**DOI:** 10.1371/journal.pone.0036567

**Published:** 2012-05-08

**Authors:** Alicia L. J. Burns, James E. Herbert-Read, Lesley J. Morrell, Ashley J. W. Ward

**Affiliations:** 1 School of Biological Sciences, University of Sydney, Sydney, New South Wales, Australia; 2 Department of Biological Sciences, University of Hull, Hull, East Yorkshire, United Kingdom; Université Paris 13, France

## Abstract

In social animal groups, an individual's spatial position is a major determinant of both predation risk and foraging rewards. Additionally, the occupation of positions in the front of moving groups is generally assumed to correlate with the initiation of group movements. However, whether some individuals are predisposed to consistently occupy certain positions and, in some instances, to consistently lead groups over time is as yet unresolved in many species. Using the mosquitofish (*Gambusia holbrooki*), we examined the consistency of individuals' spatial positions within a moving group over successive trials. We found that certain individuals consistently occupied front positions in moving groups and also that it was typically these individuals that initiated group decisions. The number of individuals involved in leading the group varied according to the amount of information held by group members, with a greater number of changes in leadership in a novel compared to a relatively familiar environment. Finally, our results show that the occupation of lead positions in moving groups was not explained by characteristics such as dominance, size or sex, suggesting that certain individuals are predisposed to leadership roles. This suggests that being a leader or a follower may to some extent be an intrinsic property of the individual.

## Introduction

In social animal groups, different spatial positions are associated with different costs and benefits [1], [2]. For example, individuals at the front of moving groups experience greater risk of predation, but also gain access to greater foraging rewards, relative to group members in other positions [3], [4], [5]. In order to balance risk against reward in this context, individuals of some fish species may move to the front of travelling groups when they are hungry and drop back into the safety of the middle positions when they are sated, thereby rotating the occupancy of the front positions among many different individuals over time [5], [6]. However, among many other social species, particularly so-called ‘restricted entry’ social groups, which are typically characterized by social hierarchies and stable group membership (e.g. primates [7]), leadership is often consistently assumed over time and across contexts by an individual or a small subset of individuals [8], [9]. For example, adults predominantly initiate group movements in groups of chacma baboons [10], rhesus macaques [11], and bar-headed geese [12]. Additionally in other such species, dominance (sheep, [13]; baboons, [14]) and sex (gorilla, [7]; musk ox [15]) of an individual are known to correlate with leadership behaviour. By contrast, so-called ‘free entry’ social groups, such as schools of fish, where group membership is not fixed, are often assumed to be egalitarian, in the sense that at any single point any group member could act as a leader and that all group members are approximately equally likely to act as a leader over time [16], [17], [18]. Nonetheless, studies have indicated some individual characteristics that may predict the spatial positioning of an individual within such a group even in these social systems [19]. For example, the largest individuals within a moving group typically occupy the front positions in fish shoals [20], [21], while individuals with a relatively bold behavioural phenotype may also be more likely to be found at the front of groups [22], [23], [24], [25].

In addition, studies have shown that occupation of positions in the front of moving groups often correlates with the initiation of group movements [26], [27]. This is not always the case, however; animals towards the rear of groups may be primarily responsible for group movements in some cases [28], [29]. A distinction must therefore be drawn between leadership in the sense of occupation of the front position of a travelling group, and leadership in the sense of initiating and determining a new group travelling direction [30], [31]. Indeed, occupying the front position of a travelling group may in some circumstances arise through an entirely passive and self-organised process, whereas initiating and determining the group's travelling direction can be seen as being a more active and intentional process [5], [31], 32,33. The motivation to act as a leader may be based in some cases on differences between group members in the amount of information that they hold about their environment, so that some individuals are relatively informed, while others are relatively naïve. In such cases, better informed individuals may assume the role of leader [34], [35], [36], [37]. An alternative scenario is that all individuals within a group have roughly the same amount of information about their environment. Under this scenario, where a group moves into a novel environment, all group members would be relatively naïve, whereas the same group operating in a more familiar environment would be relatively informed. These two conditions are likely to produce differences in the dynamics of group behaviour and leadership. In the novel environment, for example, group members may be likely to behave more cautiously, travelling more slowly and in more cohesive groups by comparison to when in the more familiar environment [38]. In addition, naïve group members are unlikely to manifest a strong directional preference, since none has information of the location of resources such as refuges or food and none has the motivation to act as a leader [39]. This in turn may lead to more frequent changes in leadership as each individual would be keen to relinquish the role and its associated costs [3].

**Figure 1:**
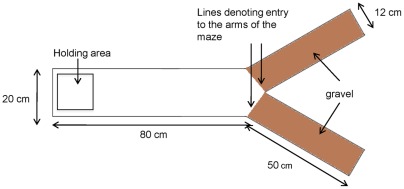
Experimental set-up.

Here we examine leadership in 18 groups of 6 mosquitofish (*Gambusia holbrooki*) as they travelled through a simple Y maze (see Fig. 1). The Y maze was used in order to allow a comparison of the spatial positioning of the fish as they moved along the stem of the Y maze in a group against their decision-making behaviour when they were at the crux of the maze. When fish reached this point where the path split into the two arms of the Y, they would then make a decision and move into one of the arms. Before the decision to move into one of the two arms was made, fish typically slowed down and increased their turning rate, behaviour which is assumed to indicate uncertainty [40], [41], [42]. This caused the shoal to bunch and allowed individuals at the back of the group the opportunity to overtake the individuals at the front of the group. This is important to note, as a fish at the front of a moving group need not necessarily be the first individual subsequently to move into one of the arms of the Y maze; the decision-making process appeared to occur once the fish had slowed, or in some cases, stopped moving forwards. Consequently, we made two separate measurements. Firstly, we measured the mean spatial position (the order from the front to the back of the travelling group) of each fish in a group as they moved through the stem of the Y maze. Secondly, we measured decision order in each group (the order in which fish entered the arm of the maze, from first to last). To examine consistency of spatial positioning and decision order in moving groups, we tested each shoal once per day for five days. Since each repeated test was carried out in the same experimental apparatus, individuals had chance to learn and become more familiar with their environment with each successive exposure. Using this approach, we were able to test four main hypotheses. Firstly, that the spatial positioning of individuals and the decision order of individuals is consistent over time. Secondly, that leadership in the sense of occupation of the front position of a travelling group is positively correlated with leadership in the sense of initiating a new group travelling direction. Thirdly, that leadership would be exchanged between group members less frequently as those group members become more familiar with their environment. Fourthly, that leadership may be determined by the relative size, sex and/or social dominance of group members.

## Materials and Methods

### Study species and husbandry

Mosquitofish (*Gambusia holbrooki*) are a freshwater fish species, introduced to Australia in the 1920's from their native North America [43]. As a study species, mosquitofish offer many advantages: they are naturally gregarious, easily obtainable and acclimate well to laboratory conditions. Mosquitofish used in this study were collected from Lake Northam, Sydney (151.1833°E, 33.8916°S) during March and April 2010. Fish were kept in 150 litre holding tanks for an initial period of one week in water temperatures consistent with their natural habitat at their time of capture (between 21.5° and 24°C) and a photoperiod of 12∶12 light: dark. Fish were fed commercial fish food twice daily (tropical gourmet flake blend, Wardley USA).

After one week in the laboratory, individuals were lightly anaesthetized sequentially using a mix of oil of cloves, water and ethanol. Each individual was then injected with a unique combination of coloured elastomer subcutaneously on their dorsal surface using a fine-gauge syringe. This allowed for subsequent individual identification. Individual measurements for length and sex were recorded at this time. After tagging, individuals were allocated to groups of 6, two weeks prior to their first trial. We constructed 9 same sex groups, and 9 mixed sex groups. Individuals remained within these groups throughout the course of the experiments. The holding time prior to the beginning of experiments was sufficient to allow the development of a basic dominance hierarchy and familiarity between group members [44], [45], [46]. Each group was maintained in a separate 75 L aquarium throughout the duration of the trials.

To identify the dominance hierarchy of each group, we observed the groups individually once per day throughout the 5-day trial period and tallied the agonistic interactions between individuals. Mosquitofish form monarchic social dominance hierarchies, where one individual assumes the role of the dominant individual, while all the others in the group are typically subordinate [47], [48]. On the basis of these observations, it was clear which individual was dominant in each group since it displayed aggression towards other individuals, while no individual displayed aggression towards it.

**Figure 2:**
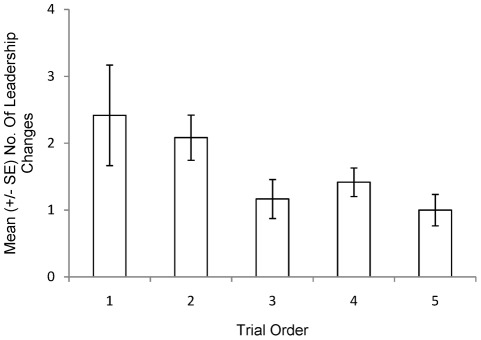
Mean number (± SE) of leadership changes as a function of trial order.

### Experimental apparatus and protocol

Trials were conducted in a Y-maze with an inclined depth range of 15 mm where the fish were released in each trial to 80 mm at the far end of the maze. The Y-maze was illuminated evenly with 23 W fluorescent lamps and surrounded by black opaque plastic to minimise any external disturbances that might influence the fish. Experimental groups of fish were placed in the holding area of the Y-maze behind a perforated gate for 90 seconds. The fish were then released by remotely lifting the gate using a monofilament line and pulley system, and were left to swim freely down the length of the Y-maze. In most cases, the fish exited the holding area within 10 seconds. Each trial was filmed using two cameras, a Canon G10 and a webcam (Quickcam Pro9000, Logitech). The Canon G10 was placed 30 cm above the holding area in order to film the groups in as they exited the holding area. This relatively close-up film was necessary to resolve each individual's tag and thus to individually identify each fish. The webcam was used to film the entire trial; it was suspended 1.2 m above the Y-maze and filmed at a frame rate of 15 Hz. We were able to identify each fish in the video taken using the webcam by cross-referencing it against the video taken using the Canon G10. Following completion of each trial, the fish were removed from the Y-maze and returned to their holding aquarium. A total of 5 trials were conducted on each group (18 groups in total) on 5 consecutive days. Each group undertook only 1 trial daily and the water in the Y-maze was changed every day. The trial order of groups was randomised each day.

### Data analysis

Videos from the webcam were converted to a stack of still images (1 per frame) using Virtual Dub v.1.9.9. These images were then imported into Image J, where we used the manual tracking facility to record the x and y coordinates of each fish throughout the trial. The position of the tip of the fish's snout was the point used for tracking. This position was recorded for each fish at every 5^th^ frame, equating to 3 sampling points per second. From the coordinate data, we calculated the spatial position of each fish in its travelling group, from front (1^st^) to back (6^th^) relative to the group's travelling direction, at each sampling point. For each trial, the number of frames that each individual spent in each position (1^st^, 2^nd^,…6^th^) was determined. We recorded positional data from the time that the fish exited the holding area of the Y-maze until the first fish entered one of the arms of the maze. We then calculated the proportion of time of each trial that an individual spent in each position of each trial and weighted these proportions separately for each fish by multiplying the proportion of time spent in 1^st^ place by 1, proportion of time spent in 2^nd^ place by 2 and so on to 6^th^ place before finally summing the values to provide a single overall value for each fish. Individuals that had occupied positions towards the front of the group had lower weighted scores than individuals that occupied positions towards the back of the group. In addition, we recorded the order in which fish crossed into the arm of the maze following their decision. An individual crossing first was given a score of 1, whilst an individual crossing last a score of 6, with sequential scores between these two positions.

### Statistical analysis

Intraclass correlation co-efficient (ICC) analysis enables the consistency of measurements across several events to be examined [49], [50]. In this case, we used the ICC to analyse whether individuals consistently occupied the same position within their group across the 5 separate trials. One ICC analysis compared the weighted spatial positions of the 6 individuals within each group across all five trials for each group. A separate ICC analysis was carried out to compare the order that fish crossed into the arm of the maze in each trial across all five trials for each group. These analyses therefore yielded two sets of statistical output for each group. In order to test if the positioning of individuals was consistent across all groups tested, we combined the p-values obtained from the ICC analyses carried out on weighted spatial position across all groups (n = 18) and, separately, combined the p-values obtained from the ICC analyses carried out on the decision order of fish in each group (n = 18) using Fisher's Omnibus procedure in each case. This provided a single global p-value for consistency of spatial positions and a single p-value for consistency of decision order across all trials of all 18 groups.

We then determined if there was a relationship between the mean weighted spatial position and the mean decision order of fish. To do this, we calculated the mean weighted spatial position of each fish in a group across all five trials and the mean decision order across all five trials for each group. We then compared these measures using a linear mixed effects model including group identity as a random factor.

To investigate the effect of trial order on the number of changes of leadership that occurred as the group moved through the apparatus from the time that the shoal left the holding area, until the first fish crossed into an arm of the maze, we used a generalised linear mixed effect model. We specified a poisson error distribution with a log link function (as appropriate to count data), and included group identity as a random effect to control for the non-independence of individuals within a group. Shoal cohesion (defined as mean distance to all group members from the group centroid when at least five group members were present within the stem of the Y-maze) conformed to the assumptions of normality (assessed by inspection of plots of model residuals), and the effect of trial number on this measure was analysed using a linear mixed effects model including group identity as a random factor. To analyse the effect of trial number on the probability that the individual that spent the most time leading the travelling group was also the individual that subsequently first decided to enter the arm of the maze, we used a generalised linear mixed effects model with a binomial error distribution and logit link function (as appropriate for this data type), and with group identity as a random factor.

To examine the effect of size on spatial positioning, we ranked each fish from within a group from 1 to 6, largest to smallest, then carried out an ANOVA with rank size as the independent variable and position as the dependent variable. We used a paired t-test to compare the mean positions of the sexes in the mixed sex groups. We also compared the overall position of the dominant fish against a null expectation of 3.5 using a single independent samples t-test. The value, 3.5, corresponds to the average position of an individual in a group of 6 that we would expect by chance over time.

## Results

### Consistency of Positioning Behaviour

Across all groups, both the spatial positioning of individuals in groups as they travelled through the main part of the maze (Fisher's Omnibus test: χ^2^_36_ = 93.6, p<0.001) and the decision order of individuals as they moved into an arm of the maze (Fisher's Omnibus test: χ^2^_36_ = 88.8, p<0.001) were highly consistent across trials.

The mean spatial positioning behaviour of the fish in each group across all trials as they travelled through the stem the maze was positively correlated with their mean decision order as they moved into the arm of the maze (Mixed effects model: t_89_ = 9.3, p<0.001).

### Leadership and spatial organization of fish in relationship to familiarity with environment

**Figure 3:**
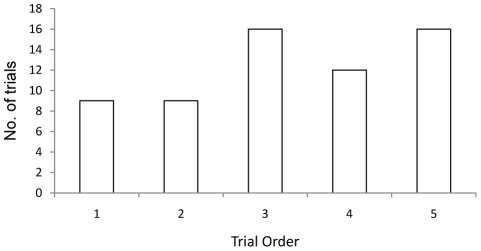
Number of trials where the individual that led the group through the maze was also the first to decide to enter an arm of the maze.

The number of changes in leadership per trial as the shoal moved through the test apparatus decreased as the number of trials that each group had undergone increased, as fish became more familiar with the environment (Mixed effects model: Z = −3.54, N = 90 in 18 groups, p<0.001; Figure 2). There was no change in shoal cohesion across the sequence of trials (Mixed effects model: t_71_ = 0.2, p = 0.846). The probability that the individual that led the travelling group was also first to make the decision to swim into one of the arms of the maze increased as the sequence of trials progressed (Mixed effects model: Z = 2.319, N = 90 in 18 groups, p = 0.02; Figure 3).

### Relationship of Positioning Behaviour to Size, Sex and Social Dominance

There was no difference between the size rank of fish in each group in terms of their mean spatial positioning across the five trials (ANOVA: F_5,60_ = 0.55, p = 0.74) or their mean decision order (ANOVA: F_5,60_ = 0.47, p = 0.79). There was no difference in the mean spatial positions of males versus females in travelling mixed sex groups (Paired t-test: t_8_ = 0.06, p = 0.95) or in terms of their mean decision order (Paired t-test: t_8_ = 1.5, p = 0.17). The mean spatial position of the dominant individual did not differ from a null expectancy of 3.5 either in a travelling shoal (Independent samples t-test: t_15_ = 0.4, p = 0.7) or in terms of its mean decision order (Independent samples t-test: t_15_ = 0.4, p = 0.7).

## Discussion

Consistency was found both in the spatial positioning of individuals as well as in their decision order, suggesting that some individuals may to an extent be predisposed either to the role of leader or the role of follower within free-entry groups, regardless of sex, size or dominance. This is in line with predictions made by Johnstone and Manica [51] regarding the evolution of intrinsic leaders and followers. They predict that when there is little conflict of interest among group members, as we assume in the present experiments, most individuals would act as intrinsic followers, with the reverse prediction for conditions of high conflict. Certain aspects of animal personality, in particular boldness, or more specifically the tendency of individuals to accept risk in return for a potential reward [23], [24], [52], may predispose individuals towards leadership. In this case, a bold individual may potentially benefit from having a disproportionate influence on group travelling direction at the cost of greater risk of predation [4], [5], [39].

An individual's spatial positioning in a travelling group and the order in which it ultimately made its decision were highly correlated, lending support to the idea that in fish shoals, those individuals in front positions have a strong influence on group travel direction; a finding consistent with a number of other studies on other fish species (roach, *Rutilus rutilus* [53], sticklebacks, *Gasterosteus aculeatus* [27], Atlantic mackerel, *Scomber scombrus* [54]) and with recent work suggesting that the information in moving shoals generally flows from front to back positions [55], [56]. Nonetheless, the likelihood that the individual that lead the group through the main part of the maze was also the one to decide first on the new travelling direction increased as the trials progressed, and as individuals presumably became more familiar with the experimental arena. This provides some support for the suggestion that leadership in terms of the simple occupation of the front positions and leadership in terms of being first to make a decision may be under slightly different constraints [5], at least in novel environments.

Leadership switched between group members more often in an unfamiliar environment than when group members had experience of that same environment. The behaviour of many animals is known to differ between familiar and unfamiliar environments, as individuals adopt risk-averse behaviour in the face of uncertainty [57]. As a result the greater frequency of leadership changes may reflect the uncertainty experienced by individuals in an unfamiliar environment; their lack of information and consequent absence of any direction preference may affect their motivation to lead. By contrast, there were far fewer changes of leadership once individuals had gained experience of the environment. In a more familiar environment, therefore, fewer individuals act as leaders. Furthermore, individuals at the front of travelling groups were also far more likely to decide on the new travelling direction in a familiar environment. This has clear implications for the collection and transmission of information throughout groups; decision-making increases in efficiency as the number of individual participating in the decision increases [40], [56], [58], [59]. In conjunction with our finding that individuals that lead the group through the maze were also likely to be the ones that initiated the new group travelling direction into an arm of the maze, we suggest that when group members have little information about their environment, more individuals may contribute to the decision-making process.

The fact that consistent leadership and spatial positioning can emerge through social interactions in these small fission-fusion groups [60] leads to intriguing questions regarding the longer-term social dynamics of larger groups. Can leader-follower interactions still exist when followers number potentially in the thousands? Or is variable leadership (high turnover for the role of leader) simply a by-product of larger group sizes and the inability of single leaders to consistently lead multiple followers? Answering these questions will provide us with a more detailed understanding of decentralised decision making processes in animal groups.
